# Anti-Fibrosis Effects of Magnesium Lithospermate B in Experimental Pulmonary Fibrosis: By Inhibiting TGF-βRI/Smad Signaling

**DOI:** 10.3390/molecules26061715

**Published:** 2021-03-19

**Authors:** Xin Luo, Qiangqiang Deng, Yaru Xue, Tianwei Zhang, Zhitao Wu, Huige Peng, Lijiang Xuan, Guoyu Pan

**Affiliations:** 1 State Key Laboratory of Drug Research, Shanghai Institute of Materia Medica, Chinese Academy of Science, 501 Haike Road, Shanghai 201203, China; luoxin17123@163.com (X.L.); qqdeng@simm.ac.cn (Q.D.); xueyaru3@simm.ac.cn (Y.X.); s19-zhangtianwei@simm.ac.cn (T.Z.); huigepeng@163.com (H.P.); 2 School of Pharmacy, University of Chinese Academy of Sciences, Beijing 100049, China; 3 School of Chinese Materia Medica, Nanjing University of Chinese Medicine, Nanjing 210033, China; zhitaowu@simm.ac.cn

**Keywords:** pulmonary fibrosis, bleomycin, magnesium lithospermate B, transforming growth factor-beta, Smad signaling

## Abstract

Pulmonary fibrosis is a severe and irreversible interstitial pulmonary disease with high mortality and few treatments. Magnesium lithospermate B (MLB) is a hydrosoluble component of *Salvia miltiorrhiza* and has been reported to have antifibrotic effects in other forms of tissue fibrosis. In this research, we studied the effects of MLB on pulmonary fibrosis and the underlying mechanisms. Our results indicated that MLB treatment (50 mg/kg) for seven days could attenuate bleomycin (BLM)-induced pulmonary fibrosis by reducing the alveolar structure disruption and collagen deposition in the C57 mouse model. MLB was also found to inhibit transforming growth factor-beta (TGF-β)-stimulated myofibroblastic transdifferentiation of human lung fibroblast cell line (MRC-5) cells and collagen production by human type II alveolar epithelial cell line (A549) cells, mainly by decreasing the expression of TGF-β receptor I (TGF-βRI) and regulating the TGF-β/Smad pathway. Further studies confirmed that the molecular mechanisms of MLB in BLM-induced pulmonary fibrosis mice were similar to those observed in vitro. In summary, our results demonstrated that MLB could alleviate experimental pulmonary fibrosis both in vivo and in vitro, suggesting that MLB has great potential for pulmonary fibrosis treatment.

## 1. Introduction

Idiopathic pulmonary fibrosis (IPF), a severe consequence of pulmonary interstitial fibrosis, is a chronic, irreversible, and fatal disease, with a median survival time of no more than 5 years [1,2,3]. Treatments for IPF therapy are extremely limited. Pirfenidone (PFD) and nintedanib are the only two effective drugs for clinical IPF medical therapies [4,5,6,7,8]. These drugs can delay, but cannot completely inhibit, the progression of this disease and cause several adverse effects (e.g., elevated liver enzymes, gastrointestinal events, and skin disorders), resulting in treatment discontinuation and adverse gastrointestinal effects [9]. Moreover, the cost of these drugs is relatively high. New drugs with significant efficacy, good tolerability, few side effects, and a low cost are urgently needed.

The main characteristics of IPF are persistent lung interstitial injury, collagen deposition, and excessive accumulation of the extracellular matrix (ECM), which eventually result in the decline of pulmonary function. Although the detailed mechanism of IPF has yet to be disclosed, previous studies have reported the roles of cytokines on pulmonary fibro-genesis, such as interleukin-4(IL-4), IL-13 and transforming growth factor-beta(TGF-β) [2,10,11,12,13]. TGF-β is one of the most crucial pro-fibrotic factors among these cytokines in tissue repair and fibrogenesis [14,15,16]. TGF-β can stimulate transdifferentiation in fibroblasts, which are converted into myofibroblasts with the upregulation of α smooth muscle actin (α-SMA) and eventually contribute to fibrotic foci in lung tissue [17,18]. On the other hand, the type II alveolar epithelial cells (AECs II) in lung tissue can also be stimulated by TGF-β to transdifferentiate into fibroblasts [19], with a loss of the epithelial phenotype and mesenchymal feature acquisition, which is known as epithelial-to-mesenchymal transition (EMT) [20]. Myofibroblasts can produce collagen, such as Col 1A1 and Col 3A1, and fibronectin, which constitute the main substances of the extracellular matrix [15,21]. The overproduction of collagen and the extracellular matrix disturbs normal physiological repair in lung tissue [22]. Inhibiting TGF-β signaling could be a potential way to treat pulmonary fibrosis [23,24,25,26].

Magnesium lithospermate B (MLB) is the major ingredient of hydrosoluble components from the traditional Chinese herb *Salvia miltiorrhiza* and has many pharmacological activities. MLB was reported to have anti-inflammatory and antioxidative effects and has been used for clinical treatments of cardiovascular diseases [27] with good safety and tolerance. Our previous research reported the protective and anti-inflammation effects of MLB on hepatic ischemia/reperfusion [28]. Other previous studies have shown that MLB might effectively attenuate liver or renal fibrosis by inhibiting the TGF-β pathway [29,30,31]. The severity and mortality of IPF patients are worse than those of patients with other forms of tissue fibrosis because of the high risk of the acute exacerbation of IPF and subsequent respiratory failure [32], and more alternatives for pulmonary fibrosis therapies will be available if MLB can effectively alleviate pulmonary fibrosis [33].

In this study, we found that MLB treatment could efficiently inhibit pulmonary fibrosis both in a bleomycin (BLM)-induced pulmonary fibrosis mouse model and in TGF-β-stimulated human type II alveolar epithelial cell line (A549) cells and human lung fibroblast cell line (MRC-5) cells. Furthermore, we revealed that the antifibrotic efficacy of MLB was mediated mainly by regulating TGF-β receptor I (TGF-βRI) and the Smad signaling pathway.

## 2. Results

### 2.1. MLB Could Alleviate Bleomycin-Induced Pulmonary Fibrosis in Mice 

**Figure 1:**
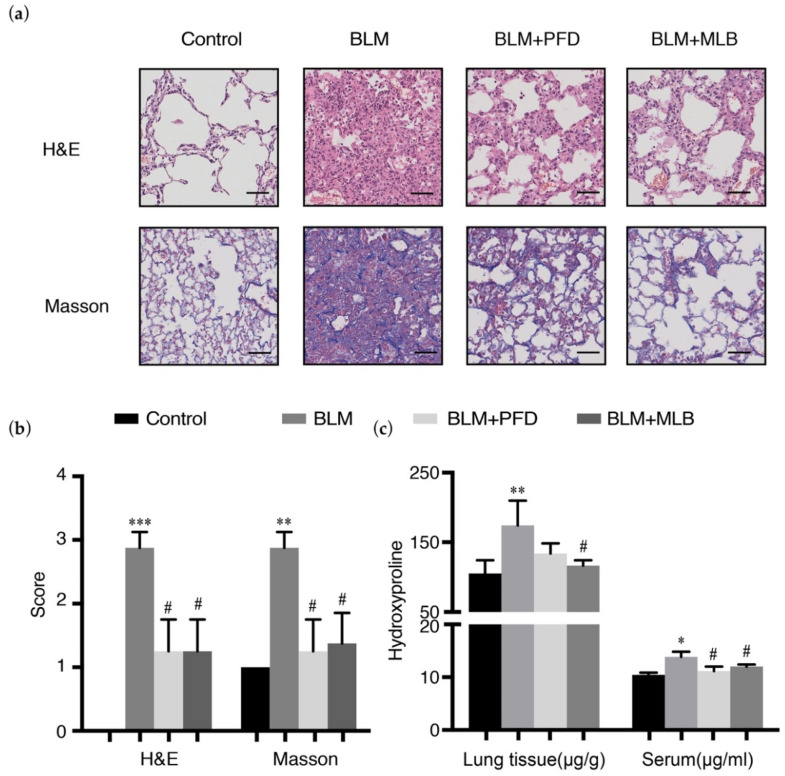
Magnesium lithospermate B (MLB) could alleviate bleomycin (BLM)-induced pulmonary fibrosis in mice. (**a**) Representative images of hematoxylin and eosin (H&E) and Masson’s trichrome-stained lung tissue slides. Bars = 50 μm, magnification: 20×. (**b**) Pathological score assessment about inflammation and fibrosis variables of each lung slice. (**c**) Total collagen content in mouse lung tissues and serum from different groups was determined by a hydroxyproline kit. Data are presented as the means ± SEM of the group and compared by one-way ANOVA. * *p* < 0.05, ** *p* < 0.01, *** *p* < 0.001 vs. Control; # *p* < 0.05, ## *p* < 0.01 vs. BLM.

The BLM treatment induced pulmonary fibrosis in a mouse model, leading to severe pulmonary injury and collagen deposition. The results of hematoxylin and eosin (H&E) and Masson’s staining revealed that bleomycin administration disrupted the alveolar spaces and lung architecture (Figure 1a), with increased collagen deposition in lung tissue, and that MLB treatment for seven days attenuated lung injury and collagen production (Figure 1a,b and Figure S1). The results of hydroxyproline measurement showed MLB could decrease the level of hydroxyproline in BLM-treated mouse lung tissue and serum (Figure 1c).

**Figure 2:**
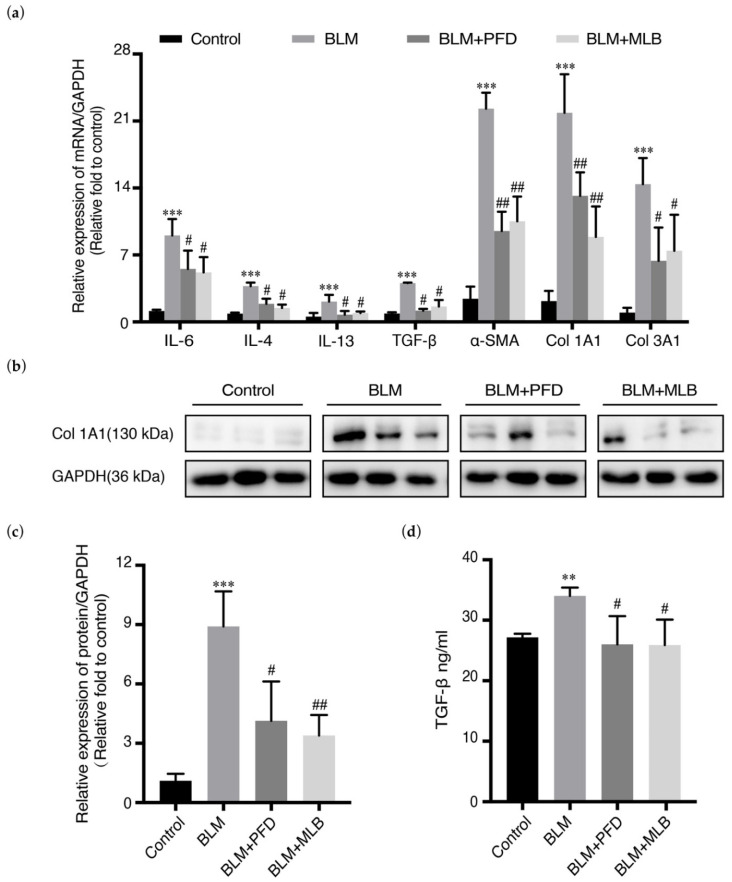
MLB decreased the expression of fibrotic genes and protein levels in BLM-treated mice. (**a**) The relative mRNA expression of interleukin (IL)-6, IL-4, IL-13, TGF-β, collagen (Col) 1A1, Col 3A1, and α smooth muscle actin (α-SMA)in mouse lung tissues from different groups was determined by real-time quantitative polymerase chain reaction (PCR). (**b,c**) The relative protein level of Col 1A1 in mouse lung tissues from different groups was determined by Western blot and analyzed by densitometry. (**d**) The level of endogenous transforming growth factor-beta (TGF-β) in serum from different groups was measured using an enzyme linked immunosorbent assay (ELISA) kit. Data are presented as the means ± SEM of the group and compared by one-way ANOVA. ** *p* < 0.01, *** *p* < 0.001 vs. Control; # *p* < 0.05, ## *p* < 0.01 vs. BLM.

The relative mRNA expression of Col 1A1, α-SMA and Col 3A1 in mouse lung tissues was increased after BLM administration, while MLB treatment significantly reduced the number of these transcripts. The relative mRNA expression of inflammatory and pro-fibrotic cytokines, such as IL-4, IL-6, IL-13, and TGF-β, could also be inhibited with MLB treatment, verifying the anti-inflammatory effects of MLB (Figure 2a). The results of Western blot analysis and enzyme linked immunosorbent assay (ELISA) further confirmed that MLB decreased BLM-induced upregulation of Col 1A1 (Figure 2b,c), and released TGF-β in serum (Figure 2d), which was consistent with the results of the mRNA expression and hydroxyproline measurement.

These results suggested that MLB could attenuate BLM-induced pulmonary fibrosis in mice. 

### 2.2. MLB Could Inhibit the Expression of Col 1A1 and TGF-β Release in A549 Cells 

**Figure 3:**
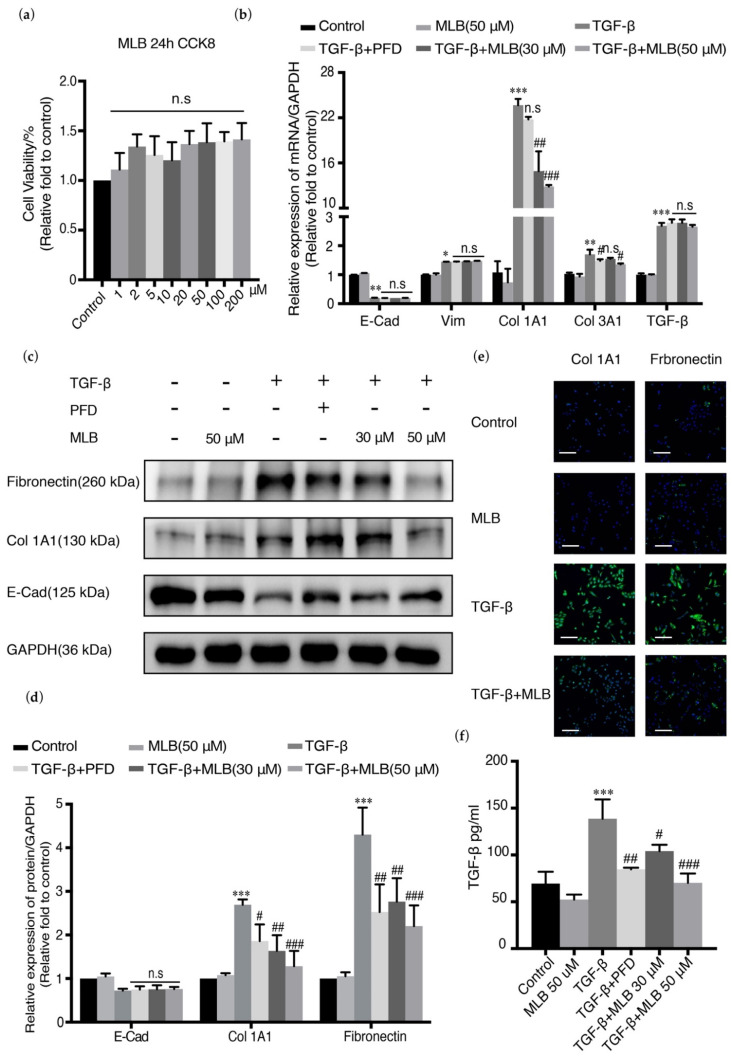
MLB inhibited collagen production in A549 cells. Quiescent cells were treated with MLB alone (50 μM), TGF-β, or both TGF-β and MLB (30, 50 μM) or pirfenidone (PFD) (30 μM) for 24 h. (**a**) Cell toxicity of MLB was measured by Cell Counting Kit-8 (CCK-8). (**b**) Relative mRNA expression of Col 1A1, Col 3A1, E-cadherin (E-Cad) and vimentin (Vim) was determined by real-time quantitative PCR. (**c**,**d**) Relative protein levels of Col 1A1, fibronectin and E-Cad were determined by Western blot and analyzed by densitometry. (**e**) Immunofluorescence staining for Col 1A1 and fibronectin. Bars = 130 μm, magnification: 4×. (**f**) The release of endogenous TGF-β was measured by using an ELISA kit. Four replicates were assessed and analyzed in each group. Data are presented as the means ± SEM of the group and compared by one-way ANOVA. The experiments were replicated four times; * *p* < 0.05, ** *p* < 0.01, *** *p* < 0.001 vs. Control; n.s *p* > 0.05, # *p* < 0.05, ## *p* < 0.01, ### *p* < 0.001 vs. TGF-β.

TGF-β plays an essential role in inducing the EMT of lung type II alveolar epithelial cells into fibroblasts, which constitutes one of the main sources of fibroblast foci in the lung and helps to promote collagen production and extracellular matrix deposition [34]. A549 cells are usually used for pulmonary fibrosis research and also used as our lung type II alveolar epithelial cells in our study [35,36,37,38]. TGF-β could induce EMT and increase the expression of collagen in A549 cells, with the downregulation of cadherin 1 (CDH-1) and upregulation of vimentin (Vim) and collagen (Col 1A1 and Col 3A1). A549 cells were treated with MLB alone, TGF-β alone, or TGF-β combined with PFD/MLB to evaluate the anti-fibrosis effect of MLB. CDH-1, Vim, Col 1A1, and Col 3A1 were employed as biomarkers. Cell Counting Kit-8 (CCK-8) analysis showed that MLB had no toxicity on A549 cells from 0 to 200 μM (Figure 3a). Real-time polymerase chain reaction (PCR) showed that TGF-β treatment substantially upregulated the relative expression of the fibrotic genes Col 1A1, Col 3A1, Vim, and TGF-β by 2200%, 170%, 140%, and 280%, respectively, with 90% downregulation of CDH1 expression compared with the control group in A549 cells (Figure 3b). The mRNA levels of Col 1A1 and Col 3A1 could be substantially and dose-dependently downregulated with MLB treatment, but the mRNA expression levels of CDH1, vimentin, and TGF-β were not changed (Figure 3b and Figure S2A). The translation of Col 1A1 and fibronectin was also inhibited by MLB according to Western blot analysis, which is consistent with the results of the cell immunofluorescence staining assay (Figure 3c–e). MLB could not reverse the downregulation of the protein level of E-cadherin (E-Cad, epithelial cell surface marker encoded by CDH1) (Figure 3c,d) compared to the TGF-β group. The release of TGF-β in A549 cells could be induced by exogenous TGF-β, which was inhibited by MLB treatment (Figure 3f). These results showed that MLB could inhibit collagen production and TGF-β release in A549 cells.

### 2.3. MLB Could Inhibit TGF-β-Induced Myofibroblast Transdifferentiation in MRC-5 Cells 

**Figure 4:**
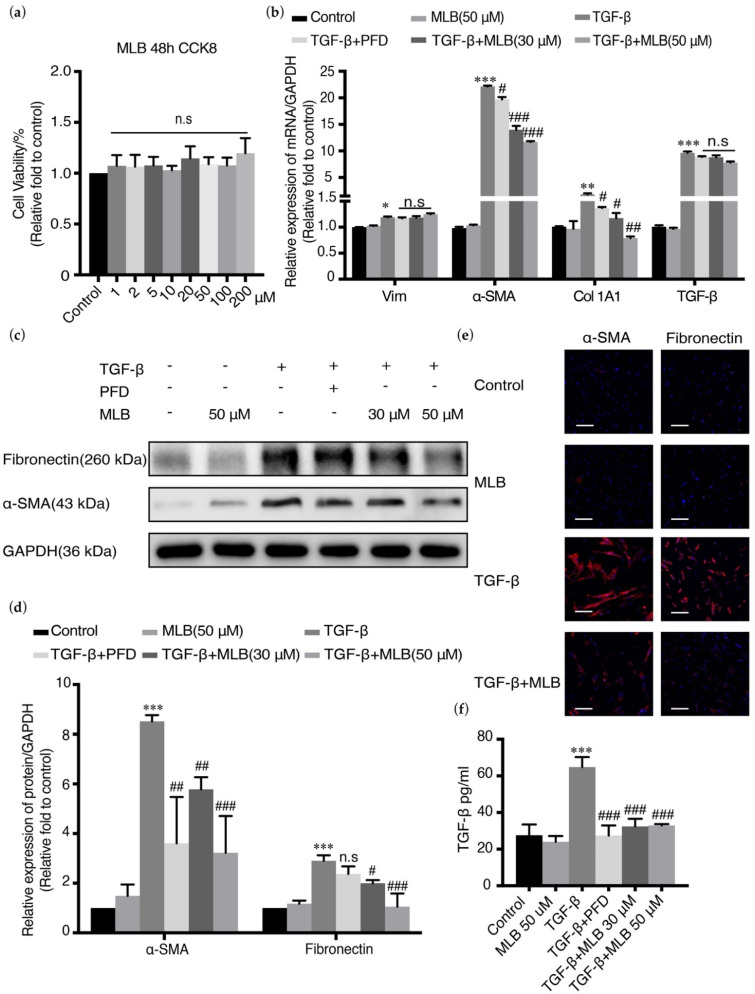
MLB inhibited TGF-β-induced myofibroblast transdifferentiation in human lung fibroblast cell line (MRC-5) cells. Quiescent cells were treated with MLB (50 μM) alone, TGF-β, or both TGF-β and MLB (30, 50 μM) or PFD (30 μM) for 48 h. (**a**) Cell toxicity of MLB was measured by CCK 8. (**b**) Relative mRNA expression of Vim, α-SMA, fibronectin, and TGF-β was determined by real-time quantitative PCR. (**c**,**d**) Relative protein levels of α-SMA and fibronectin were determined by Western blot and analyzed by densitometry. (**e**) Immunofluorescence staining for α-SMA and fibronectin. Bars = 130 μm, magnification: 4×. (**f**) The release of endogenous TGF-β was measured using an ELISA kit. Four replicates were assessed and analyzed in each group. Data are presented as the means ± SEM of the group and compared by one-way ANOVA. The experiments were replicated four times; * *p* < 0.05, ** *p* < 0.01, *** *p* < 0.001 vs. Control; n.s *p* > 0.05, # *p* < 0.05, ## *p* < 0.01, ### *p* < 0.001 vs. TGF-β.

TGF-β could also directly induce the myofibroblast transdifferentiation of lung fibroblasts, which constitutes the main resource of fibroblast foci in the lungs and regulates collagen production and ECM deposition. TGF-β could induce the upregulation of α-SMA, Vim, and fibronectin in MRC-5 cells. MRC-5 cells were treated with TGF-β in the presence or absence of MLB/PFD. The biomarkers α-SMA, Col 1A1, and fibronectin were detected. The CCK-8 analysis showed that MLB had no toxicity on MRC-5 cells (Figure 4a). Real-time PCR showed that TGF-β could upregulate the relative expression of the fibrotic genes vimentin, α-SMA, Col 1A1, and TGF-β by 120%, 2400%, 160%, and 900%, respectively, compared with those in the control group of MRC-5 cells, while the mRNA expression of α-SMA and Col 1A1 could be substantially and dose-dependently downregulated by MLB administration. MLB could not decrease the number of transcripts of vimentin and TGF-β (Figure 4b and Figure S2B). Western blot analysis demonstrated that the translation of α-SMA and fibronectin were downregulated by MLB treatment compared with that in cells treated with TGF-β alone (Figure 4c,d), which was also the same as the results of the cell immunofluorescence staining assay (Figure 4e). The release of TGF-β induced by exogenous TGF-β was also inhibited by MLB treatment in MRC-5 cells (Figure 4f). This result suggests that MLB could inhibit TGF-β-induced myofibroblast transdifferentiation in MRC-5 cells.

### 2.4. MLB Could Inhibit TGF-βRI/Smad Signaling in TGF-β-Stimulated A549 and MRC-5 Cells

**Figure 5:**
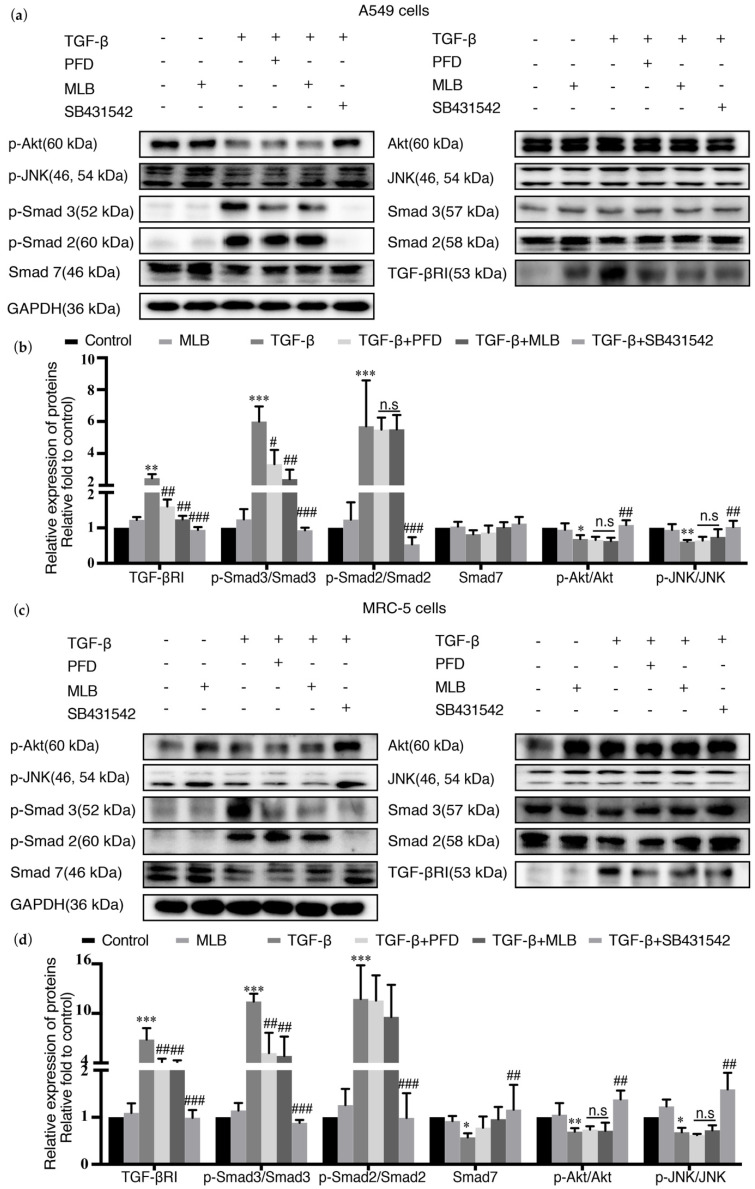
MLB inhibited TGF-β receptor I (TGF-βRI)/Smad signaling in A549 cells and MRC-5 cells. Quiescent cells were treated with MLB (50 μM) alone, TGF-β, or both TGF-β and MLB (50 μM), PFD (50 μM) and SB431542 (20 μM) for 24 h (A549 cells) or 48 h (MRC-5 cells). (**a**,**b**) The relative protein levels of TGF-βRI, Smad2, phosphorylation (p)-Smad2, Smad3, p-Smad3, Smad7, c-Jun N-terminal kinase (JNK), p-JNK, Akt, and p-Akt in A549 cells. (**c**,**d**) The relative protein levels of TGF-βRI, Smad2, p-Smad2, Smad3, p-Smad3, Smad7, JNK, p-JNK, Akt, and p-Akt in MRC-5 cells. Levels of protein were determined by Western blot and analyzed by densitometry. Four replicates were assessed and analyzed in each group. Data are presented as the means ± SEM of the group and compared by one-way ANOVA. The experiments were replicated four times; * *p* < 0.05, ** *p* < 0.01, *** *p* < 0.001 vs. Control; n.s *p* > 0.05, # *p* < 0.05, ## *p* < 0.01, ### *p* < 0.001 vs. TGF-β.

TGF-β can induce and regulate tissue fibrogenesis by activating TGF-β signaling, including the Smad pathway and non-Smad pathway. In the Smad pathway, TGF-β can increase the level of TGF-β receptor I (TGF-βRI) and the phosphorylation of Smad2/3 (p-Smad 2/3) to activate and increase fibrosis production. Smad7 is an antagonist in the Smad pathway, and it can be inhibited by TGF-β stimulation. The TGF-β inhibitor SB431542 was used as the positive treatment for this experiment. We explored the effect of MLB on the expression of TGF-βRI, the real-time (RT) PCR results showed that the mRNA expression of TGF-βRI was not changed after MLB treatment (Figure S3), but that MLB could decrease the protein level of TGF-βRI and Smad3 phosphorylation, suggesting that MLB could inhibit the TGF-β/Smad3 pathway in both A549 cells (Figure 5a,b) and MRC-5 cells (Figure 5c,d). We observed that MLB could not inhibit Smad2 phosphorylation. We found that the downregulated Smad7 might be slightly recovered with MLB treatment, but with no obvious significance. Moreover, the effects of MLB on TGF-β/c-Jun N-terminal kinase (JNK) and TGF-β/protein kinase B(PKB, usually named as Akt) signaling were also studied. We noticed that TGF-β potently decreased the Akt and JNK phosphorylation and there was no obvious effect of MLB on TGF-β-induced Akt and JNK dephosphorylation, while it could be inhibited by the TGF-β inhibitor SB431542.

This result suggested that MLB could inhibit TGF-βRI/Smad signaling in TGF-β-stimulated A549 and MRC-5 cells.

### 2.5. MLB Could Inhibit TGF-β/Smad Signaling in BLM-Treated Mice

**Figure 6:**
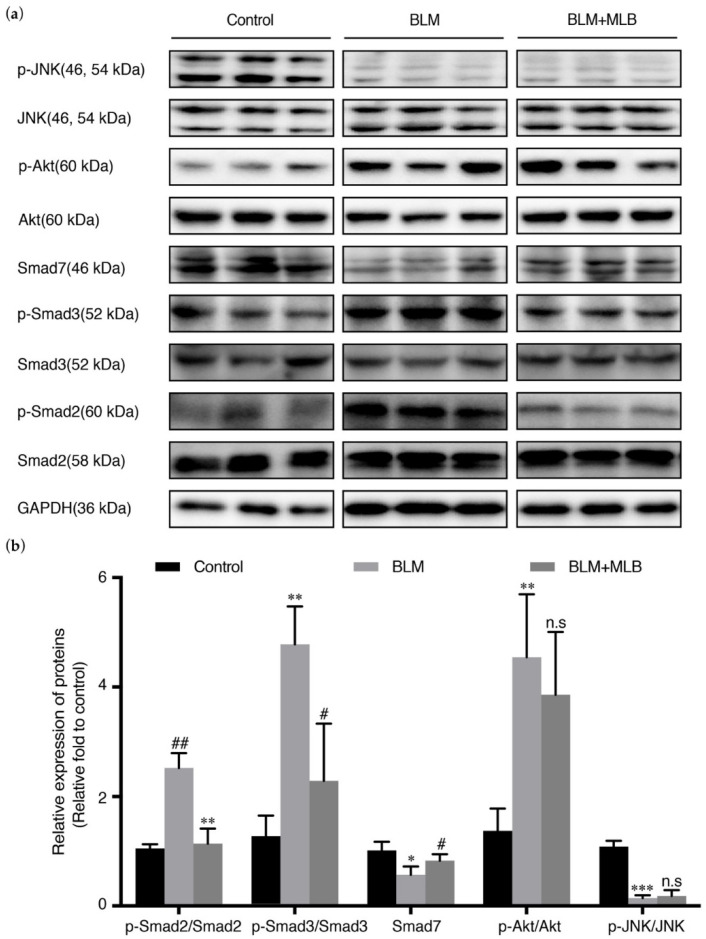
Effect of MLB on TGF-β signaling in BLM-treated lung tissue. (**a**) The relative protein levels of Smad2, p-Smad2, Smad3, p-Smad3, Smad7, JNK, p-JNK, Akt, and p-Akt in lung tissue were determined by Western blot and analyzed by densitometry (**b**). Data are presented as the means ± SEM of the group and compared by one-way ANOVA; * *p* < 0.05, ** *p* < 0.01, *** *p* < 0.001 vs. Control; n.s *p* > 0.05, # *p* < 0.05, ## *p* < 0.01, ### *p* < 0.001 vs. BLM.

TGF-β signaling was also activated in BLM-treated mice, with increased phosphorylation of Smad2, Smad3, and Akt, and downregulation of Smad7. The results of MLB on BLM-treated mice also showed the same trends: the level of Smad3 phosphorylation and release of TGF-β in serum could be decreased by MLB treatment; we also observed that MLB decreased Smad2 phosphorylation and recovered the downregulation of Smad7, but MLB still could not affect the level of JNK and Akt phosphorylation (Figure 6a,b).

These results showed that MLB treatment could inhibit TGF-β/Smad signaling in BLM-treated lung tissue.

## 3. Discussion

Current knowledge indicates that pulmonary fibrosis is mediated by myofibroblasts. The development and exacerbation of pulmonary fibrosis are characterized by the increase of fibroblast-myofibroblast transdifferentiation, accumulation, and deposition of both collagen and the extracellular matrix [2,21,39], which cause abnormal tissue repair and constant lung injury, and eventually lead to an irreversible decline in pulmonary function [14,22,32]. TGF-β plays an indispensable and pivotal role in this process, as it can directly activate myofibroblasts and regulate collagen and extracellular matrix production. Therefore, the inhibition of TGF-β signaling has received attention as a potential and useful therapy for pulmonary fibrosis [40,41]. Magnesium lithospermate B (MLB) has been reported to have anti-inflammatory, antioxidative and antifibrotic effects, especially in experimental models of liver fibrosis and renal or cardiovascular diseases, with great safety and tolerance [27,30,42]. More alternatives to treatments for pulmonary fibrosis will be provided if MLB is validated for alleviating pulmonary fibrosis. 

In our studies, we explored the effects of MLB on experimental pulmonary fibrosis. We found that MLB could attenuate lung injury and collagen deposition in a BLM-treated mouse. MLB also inhibited expressions of some inflammation or pro-fibrotic cytokines, such as interleukin and TGF-β (Figure 1 and Figure 2). From these results, we preliminary speculated that MLB might have the same effects with PFD, via the inhibition of TGF-β, contributing to pulmonary fibrosis treatments.

Myofibroblasts are the main producers and regulators of the collagen and extracellular matrix metabolism and play an important role in the maintenance of lung homeostasis, wound healing, and fibrogenesis. In lung tissue, the resources of myofibroblasts are mainly from resident lung fibroblasts, transdifferentiation of epithelial cells or endothelial cells, and migration of marrow fibroblasts [43,44]. The epithelial cells in lung tissue are divided mainly into two types: type I alveolar epithelial cells (AECs I) and type II alveolar epithelial cells (AECs II) [40,45]. AECs I mainly participate in regulating respiratory function and alveolar gas exchange, and they can get injured by pathogenic bacteria, virus, or other physical or chemical agents, causing acute alveolar destruction, lung injury, and inflammation, where some cytokines, such as IL-4, IL-13, and TGF-β, can be produced and released to promote tissue repair. Generally, AECs II can be induced to convert into AECs I for lung tissue repair, but in lung tissue with pulmonary fibrosis, owing to some unclear mechanisms, this process is abnormal, causing the transdifferentiation of AECs II into fibroblasts, which is also known as epithelial-mesenchymal transition (EMT) and contributes to myofibroblast activation [45,46,47,48]. EMT was usually reported in cancer metastasis, but it was also found to participate in tissue fibrogenesis [19,20,34,35]. Previous studies showed that EMT occurs and plays an essential role in pulmonary fibrogenesis, and that inhibiting EMT could have beneficial effects on pulmonary fibrosis treatment [26,38,49,50]. In our studies, we selected A549 cells and MRC-5 cells to evaluate the in vitro effects of MLB. As is shown in Figure 3 and Figure 4, the expression of E-Cad or α-SMA was downregulated or upregulated after TGF-β stimulation (Figure 3b–e and Figure 4b–e), indicating that the TGF-β induced EMT or myofibroblastic activation in A549 or MRC-5 cells. Our in vitro results found that MLB not only significantly inhibited TGF-β-induced MRC-5 cell myofibroblast transdifferentiation, but also decreased the production of Col 1A1 and fibronectin in TGF-β-induced A549 cells without obvious cell toxicity (Figure 3 and Figure 4). The results of cell immunofluorescence staining in A549 and MRC-5 cells showed the same inhibitory tendency of MLB (Figure 3e and Figure 4e). We observed that neither MLB nor PFD could reverse EMT in A549 cells, and that the mRNA expression of TGF-β was not influenced by MLB treatment in A549 cells or MRC-5 cells; however, from our ELISA results, we found that MLB could significantly decrease the release of endogenous TGF-β in both A549 cells and MRC-5 cells (Figure 3f and Figure 4f). These results showed the in vitro anti-fibrosis effects of MLB on alveolar epithelial cells and lung fibroblasts. 

**Figure 7:**
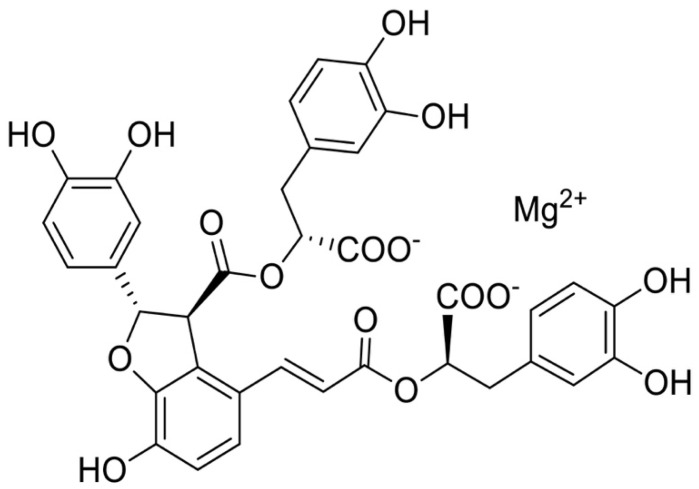
The chemical structure of MLB.

TGF-β is secreted mainly from immune cells by IL-4 or IL-13 stimulation [51,52], and it can also be released from TGF-β-activated type II alveolar epithelial cells, fibroblasts, and the extracellular matrix [21,53,54,55]. Transforming growth factor-beta receptor I (TGF-βRI) is integrated with transforming growth factor-beta receptor II (TGF-βRII) to form a complex that can recognize and bind ligands (TGF-β). TGF-β stimulation can upregulate the mRNA and protein expression of TGF-βRI and induce intracellular peptide phosphorylation of TGF-βRI to activate intracellular signal transduction [36], including that of the Smad pathway and non-Smad pathway [40]. In the TGF-β/Smad pathway, the phosphorylation of Smad 2/3 is elevated by activated TGF-βRI, and then integrates with Smad 4 to form the Smad 2/3-4 complex. Smad 7 is an antagonist in the Smad pathway because it can inhibit the phosphorylation of Smad 2/3 and the formation of the Smad 2/3-4 complex, and it can be downregulated by TGF-β stimulation. The Smad 2/3-4 complex then translocates into the nucleus to act as a transcription factor, leading to the upregulation of fibrosis transcripts, such as α-SMA, Col 1A1, Col 3A1, TGF-β, and fibronectin [56,57,58]. TGF-β can also directly activate non-Smad pathways, including mitogen-activated protein kinase (MAPK)/JNK, MAPK/p38, phosphatidylinositol 3-kinase (PI3K)/Akt and Rho-like GTPase [59,60]. These pathways could also regulate fibrosis directly or indirectly [61,62]. Previous studies indicated that the TGF-β/Smad pathway was the most important pathway involved in the pathogenesis of tissue fibrosis [56,63]. Inhibiting TGF-βRI and Smad signaling could significantly attenuate pulmonary fibrosis [23,26,49,64,65]. Our results showed that MLB could decrease the secretion of TGF-β from A549 cells and MRC-5 cells (Figure 3f and Figure 4f). Moreover, we found that MLB could reduce TGF-β-induced TGF-βRI protein levels in A549 and MRC-5 cells, with no significant effect on TGF-βRI mRNA expression (Figure S2 and Figure 5). MLB decreased Smad 3 phosphorylation but had no obvious effect on Smad 2 phosphorylation in the two cell models. The expression of Smad 7 was decreased after TGF-β stimulation in A549 and MRC-5 cells, and we observed that MLB might slightly recover the level of Smad 7 (Figure 5). In BLM-treated mouse lung tissues, MLB significantly decreased Smad 2 and Smad 3 phosphorylation and recovered the downregulation of Smad 7 (Figure 6), showing the inhibitory effect of MLB on TGF-β/Smad signaling. It was found that the treatment with MLB decreased Smad 3 phosphorylation but had no effect on Smad 2 phosphorylation in the in vitro cell culture studies. However, in BLM-treated mouse lung tissues, MLB significantly decreased both Smad 2 and Smad 3 phosphorylation. The in vitro-in vivo correlation (IVIVC) disconnection may be due to the different concentrations needed to inhibit Smad 2 and Smad 3 phosphorylation in vitro. We speculated that MLB might inhibit Smad 2 phosphorylation with higher concentrations in TGF-β-stimulated A549 and MRC-5 cells, and this should be further explored. We also studied the effect of MLB on TGF-β-induced Akt and JNK signaling. In JNK signaling, we observed that TGF-β treatment for 24 h decreased the JNK phosphorylation in TGF-β-induced A549 and MRC-5 cells or BLM-treated mouse lung tissues (Figure 5 and Figure 6), which was inconsistent with previous studies about TGF-β/JNK signaling [59,66,67,68,69,70]. Interestingly, SB431542, the potent inhibitor to TGF-β, was found to recover the downregulated JNK phosphorylation in A549 and MRC-5 cells (Figure 5). In Akt signaling, the in vitro results showed TGF-β treatment for 24 h also decreased the Akt phosphorylation (Figure 5), different from several previous results about TGF-β/Akt signaling [18,71,72,73,74]. However, there were also some studies indicating that the inhibitory or promoting effect of TGF-β on Akt phosphorylation depended on different treatment times, and the phenomena about Akt antagonizing Smad 3 [59,75]. We also observed that the inhibitory effect of TGF-β on Akt signaling was reversed by SB431542 treatment (Figure 5). We speculated that TGF-β-induced dephosphorylation of Akt was related to upregulated Smad 3 phosphorylation. We observed that MLB could not affect JNK and Akt phosphorylation in TGF-β-stimulated A549 and MRC-5 cells and BLM-treated mouse lung tissues (Figure 5 and Figure 6), indicating that the anti-fibrosis effect of MLB may not rely on JNK and Akt signaling. We noticed that MLB could only decrease the protein level of TGF-βRI, with no influence on mRNA expression (Figure S2 and Figure 7), suggesting that MLB might affect the production or degradation of TGF-βRI translation. Previous studies reported that some growth factors or proteins, like fibroblast growth factor-1 (FGF-1) or caveolin-1, could inhibit pulmonary fibrosis by regulating the degradation of TGF-βRI [23]. TGF-βRII and other non-Smad signaling pathways, like Rho-like GTPase, MAPK/p38, and MAPK/extracellular signal-regulated kinase (ERK), also play pivotal roles in pulmonary fibrogenesis [60,76,77,78,79]. The detailed effects of MLB on TGF-βRI, TGF-βRII, and other TGF-β/non-Smad pathways deserve to be clarified. Although we systematically investigated the attenuation of MLB on pulmonary fibrosis via inhibiting TGF-βRI/Smad signaling by our in vivo and in vitro models, the information obtained from our in vitro models is limited and could not completely reflect our in vivo results. For example, the in vitro models focused on fibrosis induced by TGF-β simulation alone, while the in vivo animal model included both fibrosis and inflammation symptoms induced by BLM [80,81,82]. We believed that the anti-inflammation effects and relative mechanisms of MLB on in vitro TGF-β-related inflammation models, such as IL-4/13-induced macrophages, deserve further studies.

In our research, we demonstrated that MLB treatment could attenuate BLM-induced pulmonary fibrosis in mice, TGF-β-induced A549 cell collagen production, and MRC-5 cell myofibroblast transdifferentiation, mainly by regulating TGF-βRI/Smad signaling. MLB could significantly decrease the protein levels of TGF-βRI in A549 and MRC-5 cells, and inhibited Smad signaling in vivo and in vitro, with no obvious effects on JNK and Akt pathways. We also validated the antifibrotic effects of PFD in vivo and in vitro [83,84], and based on our results, the antifibrotic effects of MLB and PFD seemed to be similar. These findings revealed that MLB, mainly by inhibiting the TGF-βRI and Smad signaling pathways, had partial anti-fibrosis effects of the TGF-β inhibitor and showed good efficacy in the treatment of pulmonary fibrosis.

## 4. Materials and Methods

### 4.1. Chemicals and Reagents

The MLB (>98% purity) was a kind gift from Xuan’s laboratory at the Shanghai Institute of Materia Medica, Chinese Academy of Science, Shanghai, China. The structure of MLB is shown in Figure 7. The F-12 Kaighn’s modification medium (F12-K) and Minimum Essential Medium/Earle’s Balanced Salt Solution(MEM/EBSS) were obtained from HyClone (South Logan, UT, USA). Fetal bovine serum (FBS), penicillin-streptomycin, TRIzol reagent, and Western Blotting electrochemiluminescence (ECL) substrates were purchased from Thermo Fisher Scientific (Waltham, MA, USA). The Hieff^®^ qPCR SYBR^®^ Green Master Mix was obtained from Yeasen Biotech Co. Ltd., Shanghai, China. Anti-glyceraldehyde-3-phosphate dehydrogenase (GAPDH), anti-p-Akt, anti-Akt, anti-p-JNK, anti-JNK, and anti-p-Smad3 antibodies were purchased from Cell Signaling Technology (Danvers, MA, USA). Anti-Col 1A1, anti-actin alpha 2 (ACTA2, α-SMA), anti-E-Cadherin (E-Cad), anti-Smad3, and anti-fibronectin antibodies were obtained from Proteintech (Wuhan, China). The anti-TGF-β receptor I (TGF-βRI) antibody was purchased from Bio-Techne R&D Systems (Minneapolis, MN, USA). Horseradish peroxidase (HRP)-conjugated secondary antibodies for rabbits, mice, or rats were obtained from Yeasen Biotech Co. Ltd., Shanghai, China. The Alexia Fluor 488^®^ secondary antibody for rabbits was purchased from Invitrogen (Waltham, MA, USA). DMSO, and the phosphatase inhibitor cocktail and bovine serum albumin (BSA) were obtained from Sigma-Aldrich (St. Louis, MO, USA). Bleomycin (BLM), pirfenidone (PFD), and trypsin (0.25%) were purchased from Meilun Biotechnology (Dalian, China). Recombinant human TGF-β1 was obtained from PeproTech (Rocky Hill, NJ, USA). The TGF-β inhibitor SB431542 was purchased from MedChem Express (Monmouth Junction, NJ, USA).

### 4.2. Bleomycin-Induced Pulmonary Fibrosis Mouse Model

C57/bl6 mice (male, ~23–25 g, 6–7 weeks old) were provided by the Experimental Animal Centre of the Shanghai Institute of Materia Medica, Chinese Academy of Science. (Shanghai, China). All the mice were fed standard food and water and kept in a temperature-controlled (~20–22 °C) Specific Pathogen Free (SPF) animal room with a 12 h light/12 h dark cycle. All experimental procedures were executed according to the guidelines for animal experimentation of the Shanghai Institute of Materia Medica, and the project was reviewed and approved by the animal ethics committee of the institution (IACUC: 2018-11-PGY-27).

The mice were injected intratracheally with bleomycin (~1.5–2.0 U/mg, 2 mg/kg, diluted in sterile saline), or an equal volume of saline as a control [31,32]. Seven days later, all alive BLM-treated mice were randomly divided into three groups (n = 8/per group) as follows: model group (BLM group), PFD treatment group (BLM+PFD group), and MLB treatment group (BLM+MLB group). Mice in the BLM+MLB group or the BLM+PFD group were given daily intraperitoneal (i.p) injections of MLB (50 mg/kg/day, dissolved in sterile saline) or an oral administration of PFD (300 mg/kg/day, dissolved in sodium carboxymethylcellulose) as the positive control [33]. All mice were euthanized 14 days after BLM administration. Serum was collected for hydroxyproline measurement and the ELISA, and lung tissues were immediately removed, washed, and cut into two parts. One part of the lung tissues was fixed in a 4% formaldehyde solution to perform histological and immunohistochemical analysis, the other was stored at −80 °C for further analysis. Pulmonary fibrosis was determined by the relative transcripts of collagen and the pro-fibrotic cytokines in lung tissue.

### 4.3. Histology Analysis and Scoring

The lung tissue slices were taken from the same site of lung tissue from all animals and were fixed in a 4% formaldehyde solution, embedded in paraffin, and subsequently stained with hematoxylin and eosin (H&E) and Masson’s staining. After staining, the inflammation and fibrosis variables of each lung slice were assessed on the basis of a blinded reported scoring system by Zuocheng Biotechnology Co., LTD, Shanghai, China [34]. At least four replicates from each group were analyzed.

### 4.4. Hydroxyproline 

Hydroxyproline is a product of proline hydroxylation and unique to all the collagens, and is also the one of major factors in collagen metabolism. The content of hydroxyproline can serve as a representative standard to evaluate the extent of tissue fibrosis [35]. The level of hydroxyproline in serum and lung tissues was measured according to a hydroxyproline assay kit (A030-1-1, Nanjing Jiancheng Bioengineering Institute, Nanjing, China). 

### 4.5. Cell Culture and Treatment

The A549 cells (human type II alveolar epithelial cell line) and MRC-5 cells (human embryonic lung fibroblast line) were kindly provided by the Stem Cell Bank, Chinese Academy of Sciences (Shanghai, China). A549 or MRC-5 cells were respectively cultured in the F-12 Kaighn’s modification medium or the MEM/EBSS medium containing 10% fetal bovine serum, 50 U/mL penicillin, and 50 μg/mL streptomycin in a humidified incubator at 37 °C with 5% CO_2_. A549 cells were passaged every 2 days; the MRC-5 medium was replaced every 2 days, and the cells were passaged every 5 days.

A549 or MRC-5 cells were seeded at a density of 2 × 10^5^ cells/mL in 6-well plates. After growing for 8 h under normal growth conditions, the cells were rendered quiescent, by incubating them in a serum-free medium for 24 h, and then treated with 10 ng/mL TGF-β1, MLB (dissolved in sterile water), both TGF-β1 and MLB, or pirfenidone(PFD, dissolved in DMSO)/SB431542 (dissolved in DMSO) for 24 h(for A549 cells) and 48 h (for MRC-5 cells). Cells treated with Phosphate Buffer Saline (PBS) or PFD/SB431542 were used as the negative or positive control. After incubation, cells were collected for further transcriptional or translational analysis. All the experiments were replicated four times.

### 4.6. Cell Viability Assay

The cytotoxicity of MLB in A549 or MRC-5 cells was assessed using a Cell Counting Kit (CCK-8, Cat No. 40203; Yeasen Biotech Co. Ltd., Shanghai, China). A549 or MRC-5 cells were seeded at a density of 2 × 10^4^ cells per well in 96-well plates. After growing for 8 h under normal growth conditions, cells were rendered quiescent by incubating in a serum-free medium for 24 h, and then treated with a normal medium containing various concentrations of MLB (0–200 μM). As the negative control, MLB was added to the medium without cells. After incubation for up to 24–48 h, 10 μL of the CCK8 solution was added to each well and incubated for ~1–2 h. The optical density at 450 nm was measured using a SYNERGY microplate reader (Biotek Winooski, Vermont, USA).

### 4.7. RNA Extraction and Real-Time Quantitative PCR (RT-qPCR) 

The total RNA was extracted from the A549 or MRC-5 cells and mouse lung tissues using TRIzol reagent, and reverse-transcribed into cDNA (Takara, Dalian, China). Quantitative real-time polymerase chain reaction (PCR) was performed on a 7500 Fast Real-Time PCR system (Applied Biosystems, Thermo Fisher Scientific; Waltham, MA, USA) using a Hieff^®^ qPCR SYBR^®^ Green Master Mix to determine the relative mRNA expression of α-SMA, Col 1A1, Col 3A1, TGF-β, E-Cad, and TGF-βRI in A549 or MRC-5 cells and mouse lung tissues. All the values were normalized against the housekeeping gene glyceraldehyde-3-phosphate dehydrogenase (GAPDH) with the delta-delta Cycle Threshold (ΔΔCT) method. The PCR primer pairs are shown in Table S1.

### 4.8. Western Blot Analysis

The total proteins from A549 or MRC-5 cells were extracted by homogenization in ice-cold RIPA buffer (Beyotime Biotechnology, Jiangsu, China), supplemented with a cocktail (1 mmol/L). The lung tissues were homogenized with liquid nitrogen and lysed in ice-cold RIPA buffer, and the supernatants of lung tissue homogenates or cell lysis solutions were obtained by centrifugation at 12,000 g for 10 min. The protein concentrations were quantified using a BCA assay kit (Beyotime Biotechnology, Jiangsu, China) according to the manufacturer’s instructions. Equal amounts of protein samples were intermixed with a sodium dodecyl sulfate (SDS)–loading buffer (Yeasen Biotech Co. Ltd., Shanghai, China) and then boiled for 10 min at 100 °C. Protein samples (20 μg/lane) were separated by 8% SDS–polyacrylamide gel electrophoresis (SDS-PAGE), blotted onto a polyvinylidene difluoride membrane (Merck Millipore, Darmstadt, Germany), blocked with 5% nonfat milk or bovine serum albumin, and incubated with primary antibodies (Table S2) at 4 °C overnight. The membranes were then washed three times with Tris-buffered saline containing 0.1% Tween-20 (TBST) and incubated with the appropriate horseradish peroxidase (HRP)-conjugated secondary antibody (1:5000) for 2 h at room temperature. After rinsing three times in TBST, the immunolabelled proteins were visualized using Western blotting ECL substrates.

### 4.9. Cell Immunofluorescence Staining 

Cells were fixed with 4% paraformaldehyde for 15 min and then incubated with 1% BSA in PBS at room temperature to block nonspecific sites for 1 h. For staining, the cells were incubated with the following rabbit polyclonal primary antibodies at 4 °C overnight: α-SMA, Col 1A1, and fibronectin (Table S2). Subsequently, the cells were washed with phosphate-buffered saline (pH = 7.2) and incubated with an Alexia Fluor 488^®^ secondary antibody. The dilution ratio for secondary antibodies was 1:1000. The cells were then incubated with 4’,6-diamidino-2-phenylindole (DAPI) (Beyotime, Shanghai, China) at room temperature for 1 h. Fluorescent images were taken using a revolve fluorescence microscope (Echo-lab, Broomfield, CO, USA).

### 4.10. Enzyme Linked Immunosorbent Assay (ELISA) 

For animals, the collected blood was stored overnight at 4 °C and then centrifuged for serum. For cells, after 24–48 h of culture, where A549 or MRC-5 cells were treated as previously mentioned, the medium was then replaced with a serum-free medium. Cell-free supernatants were collected at another 24 h. The levels of TGF-β1 were measured in supernatants or serum using human/mouse/rat TGF-β1 ELISA kits (Multi Sciences, Hangzhou, China) according to the manufacturer’s instructions. Samples with a concentration exceeding the limits of the standard curve were repeated after dilution. All of the reported experiments were performed three times. 

### 4.11. Statistical Analysis 

For each experiment, the values were expressed as the mean ± SEM. The differences among groups were statistically assessed by one-way analysis of variance (ANOVA) followed by Tukey’s multiple-comparison tests using GraphPad Prism software (version 7.0a). There was a statistically significant difference when the *p* value was less than 0.05. 

## 5. Conclusions

MLB treatment for seven days could attenuate BLM-induced pulmonary fibrosis in mice. MLB could significantly inhibit TGF-β-induced A549 cell collagen production and MRC-5 cell myofibroblast transdifferentiation, mainly by decreasing the protein level of TGF-βRI in A549 and MRC-5 cells, and inhibiting Smad signaling in vivo and in vitro, with no obvious effects on JNK and Akt pathways. These findings revealed that MLB, mainly by inhibiting the TGF-βRI and Smad signaling pathways, had partial anti-fibrosis effects of the TGF-β inhibitor and showed good efficacy in the medical treatment of pulmonary fibrosis.

## Data Availability

All datasets generated for this study are included in the manuscript and/or the Supplementary Files.

## Supplementary Materials

Figure S1: MLB had no effects in normal mice; Figure S2: MLB could dose-dependently inhibit transcripts of fibrosis in A549 cells and MRC-5 cells; Figure S3: MLB could not affect the mRNA level of TGF-βRI in TGF-β-stimulated A549 cells and MRC-5 cells. Table S1: PCR primer pairs used for research; Table S2: Information about relative antibodies used for research.

## Sample Availability

All samples of the compounds are available from all the authors.
