# Supplementary material for: Anti-Fibrosis Effects of Magnesium Lithospermate B in Experimental Pulmonary Fibrosis: By Inhibiting TGF-βRI/Smad Signaling
Source: Molecules. 2021 Mar 19;26(6):1715. doi: 10.3390/molecules26061715 (PMC8003516; doi:10.3390/molecules26061715)
Supplement: Supplementary file 1 [file molecules-26-01715-s001.pdf]

Article

Anti-Fibrosis Effects of Magnesium Lithospermate B in Experimental Pulmonary Fibrosis: By Inhibiting TGF-βRI/Smad Signaling

Xin Luo [1,2], Qiangqiang Deng [1], Yaru Xue [1,2], Tianwei Zhang [1,2], Zhitao Wu [3], Huige Peng [1], Lijiang Xuan [1,2,*] and Guoyu Pan [1,2,*]

[1] State Key Laboratory of Drug Research, Shanghai Institute of Materia Medica, Chinese Academy of Science, 501 Haike Road, Shanghai 201203, China; luoxin17123@163.com (X.L.); qqdeng@simm.ac.cn (Q.D.); xueyaru3@simm.ac.cn (Y.X.); s19-zhangtianwei@simm.ac.cn (T.Z.); huigepeng@163.com (H.P.); ljxuan@simm.ac.cn (L.X.)

[2] School of Pharmacy, University of Chinese Academy of Sciences, Beijing 100049, China

[3] School of Chinese Materia Medica, Nanjing University of Chinese Medicine, Nanjing 210033, China; zhitaowu@simm.ac.cn

* **Correspondence: ljxuan@simm.a**c.cn (L.X.); gypan@simm.ac.cn (G.P.)

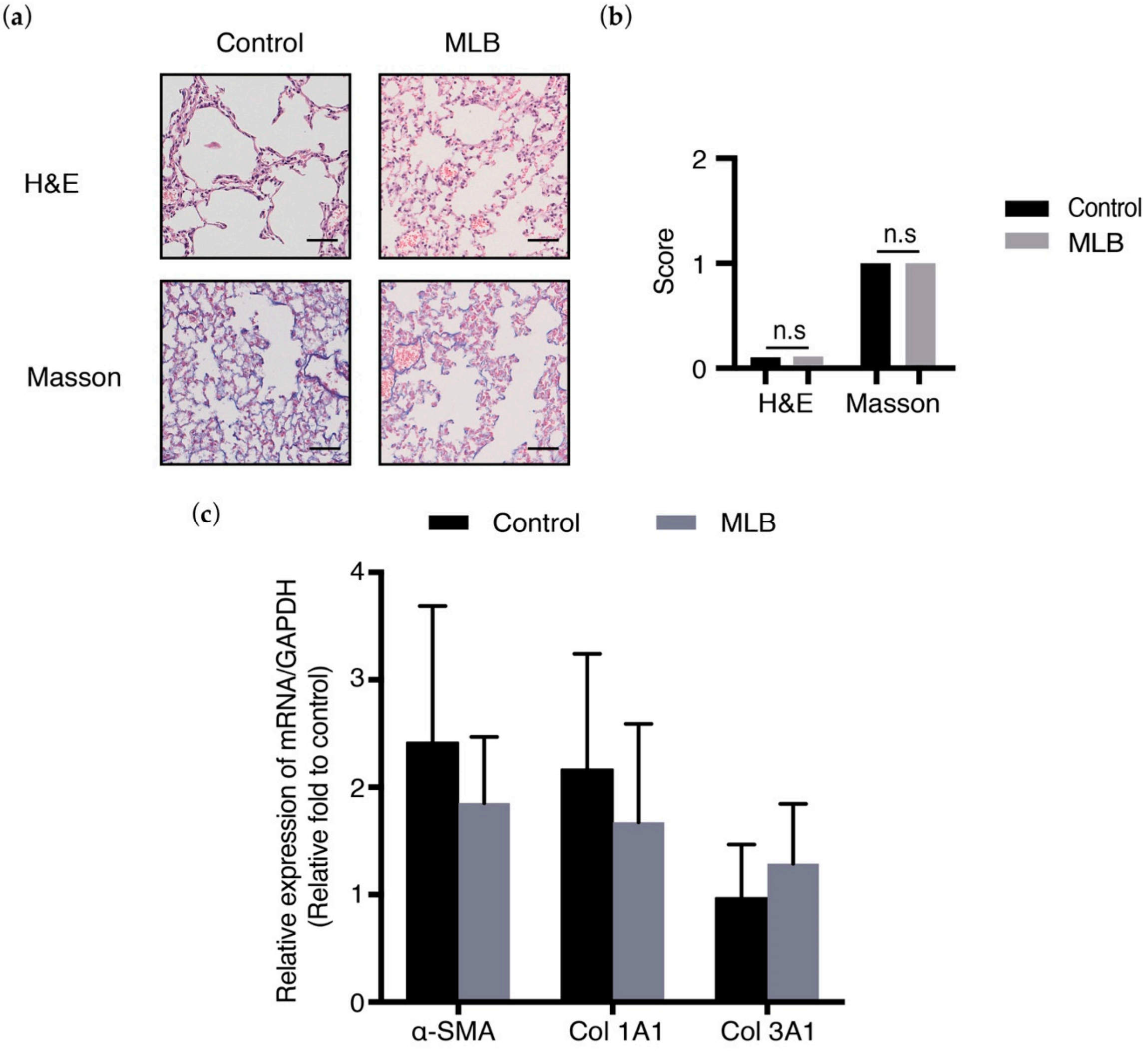

Figure S1. MLB had no effects in normal mice. mice in MLB group were treated with MLB for seven days (i.p. 50 mg/kg/day), and mice in control group were given saline(n=3). **(a)** Representative images of hematoxylin and eosin (H&E) and Masson's trichrome stained lung tissue slides. Bars = 50 μm,

magnification: 20 ×. **(b)** Pathological score assessment about inflammation and fibrosis variables of each lung slices. **(c)** Relative mRNA expression of Col 1A1, Col 3A1 and α-SMA in mouse lung tissues from different groups was determined by real-time quantitative PCR. Data are presented as the means ± SEM of the group and compared by one-way ANOVA. n.s P>0.05 vs. Control.

(**a**)

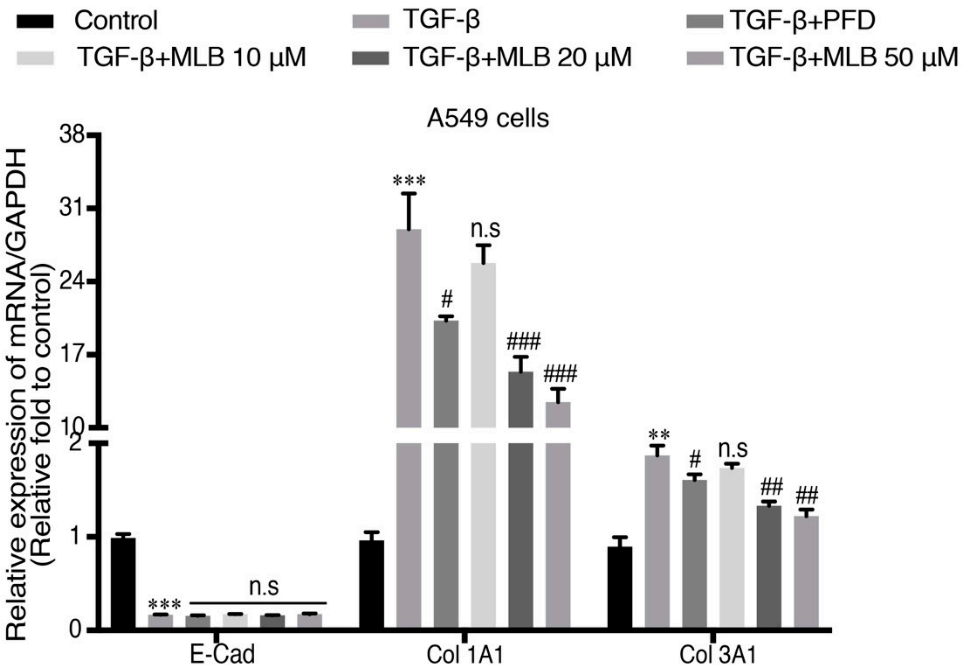

(**b**)

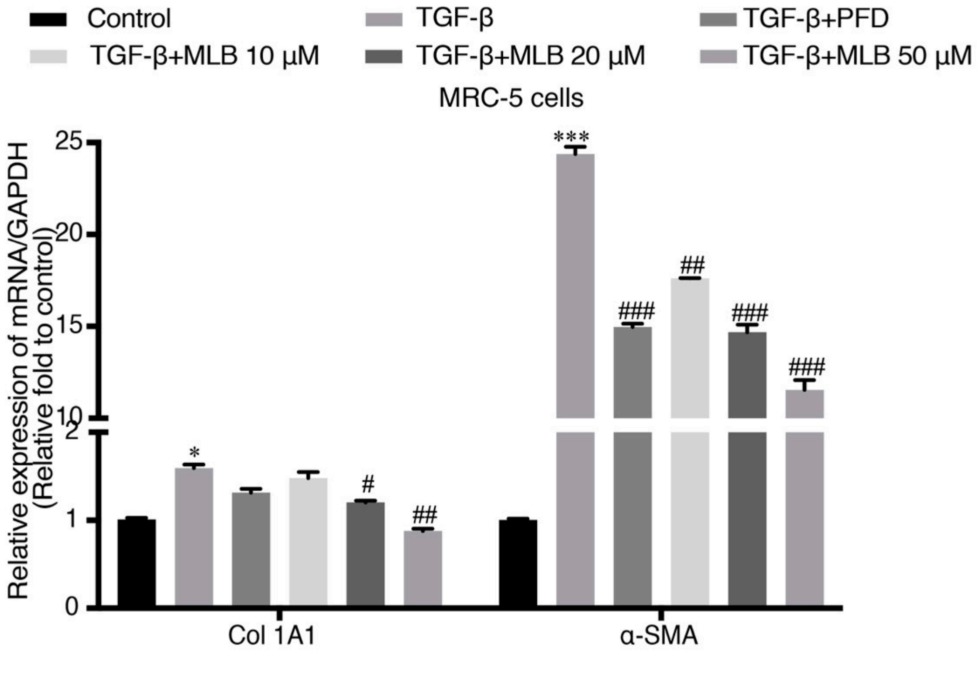

Figure S2. MLB could dose-dependently inhibit transcripts of fibrosis in A549 (a) cells and MRC-5 (b) cells. Quiescent cells were treated with TGF-β or both TGF-β and MLB with different dosages for 24 h (A549 cells) or 48 h (MRC-5 cells). Data are presented as means ± SEM of the group and compared by One-Way ANOVA (n = 4); *P < 0.05, **P < 0.01, ***P < 0.001 vs. Control; n.s P>0.05, #P < 0.05, ##P < 0.01, ###P < 0.001 vs. TGF-β.

(**a**) (**b**)

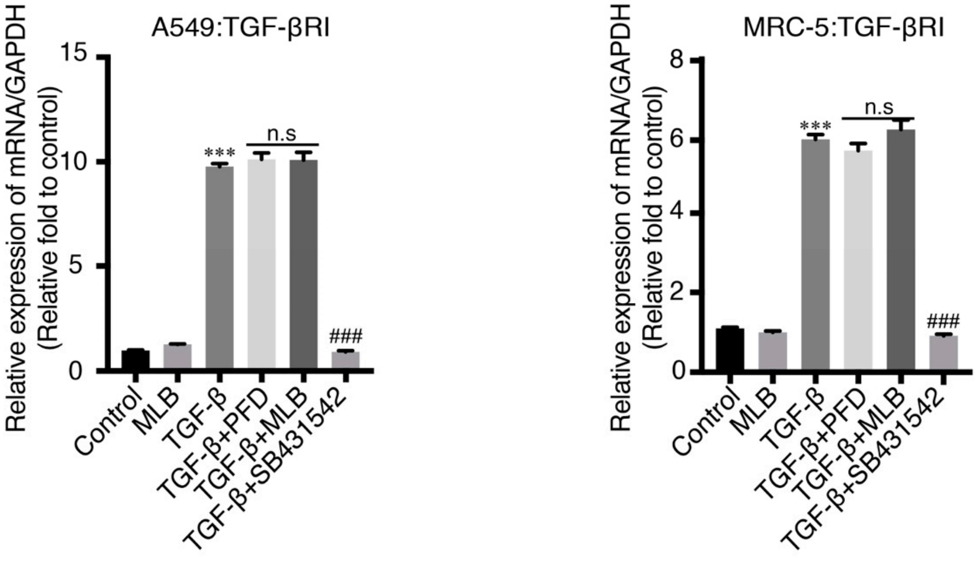

Figure S3. MLB could not affect the mRNA level of TGF-βRI in TGF-β stimulated A549 cells(a) and MRC-5 cells(b). Quiescent cells were treated with MLB (50 μM) alone, TGF-β, or both TGF-β and MLB (50 μM), PFD (50 μM) and SB431542 (20 μM) for 24 h (A549 cells) or 48 h (MRC-5 cells).Data are presented as means ± SEM of the group and compared by One-Way ANOVA (n = 4); ***P < 0.001 vs. Control; n.s P>0.05, ###P < 0.001 vs. TGF-β.

Table S1. PCR primer pairs used for research (H: Human; M: Mice).

Primer sequence		
H-α-SMA	Forward	5′- ATG CTC CCA GGG CTG TTT TC-3′
	Reverse	5′-CTT TTG CTC TGT GCT TCG TC-3′
H-CDH 1	Forward	5′- GAG AAC GCA TTG CCA CAT ACA C-3′
	Reverse	5′- GAG CAC CTT CCA TGA CAG ACC C-3′
H-Col 1A1	Forward	5′- TGA CGA GAC CAA GAA CTG CC-3′
	Reverse	5′- GCA CCA TCA TTT CCA CGA GC-3′
H-Col 3A1	Forward	5′- CGC CCT CCT AAT GGT CAA GG-3′
	Reverse	5′- TTC TGA GGA CCA GTA GGG CA-3′
H-GAPDH	Forward	5′- AAG AAG GTG GTG AAG CAG G-3′
	Reverse	5′- AGG TGG AGG AGT GGG TGT CG-3′
H-TGF-β1	Forward	5′- CAG CAA CAA TTC CTG GCG ATA-3′
	Reverse	5′- GCT AAG GCG AAA GCC CTC AAT-3′
H-TGF-βRI	Forward	5′-ACT TCC AAC TAC TGG CCC TT-3′
	Reverse	5′-ATG GTG AAT GAC AGT GCG GT-3′
H-Vimentin	Forward	5′- TGC GTG AAA TGG AAG AGA ACT-3′
	Reverse	5′- TCA GGT TCA GGG AGG AAA AGT-3′
M-α-SMA	Forward	5′- GTT TCG GGA GCA GAA CAG AGG-3′
	Reverse	5′- GAA GCT GGC CGT TCA CTC TA-3′
M-Col 1A1	Forward	5′- CAA TGG CAC GGC TGT GTG CG-3′
	Reverse	5′- AGC ACT CGC CCT CCC GTC TT-3′
M-Col 3A1	Forward	5′- GAG GAA TGG GTG GCT ATC CG-3′
	Reverse	5′- TTG CGT CCA TCA AAG CCT CT-3′
M-GAPDH	Forward	5′-AGG TCG GTG TGA ACG GAT TTG-3′
	Reverse	5′- GGG GTC GTT GAT GGC AAC A-3′
M-TGF-β1	Forward	5′- CCA CCT GCA AGA CCA TCG AC-3′
	Reverse	5′- CTG GCG AGC CTT AGT TTG GAC-3′
M-IL-4	Forward	5′-ATG GAT GTG CCA AAC GTC CT-3′
	Reverse	5′-AAG CAC CTT GGA AGC CCT AC-3′
M-IL-6	Forward	5′-CCT ACC CCA ATT TCC AAT GCT C-3′
	Reverse	5′-GGT CTT GGT CCT TAG CCA CT-3′

M-IL-13	Forward	5′-GAA TCC AGG GCT ACA CAG AAC-3′
	Reverse	5′-AAC ATC ACA CAA GAC CAG ACT C-3′

Table S2. Information about relative antibodies used for research. (WB: Western blot analysis; IF: Cell immunofluorescence staining; H: human; M: mouse; R: rat.).

Antidody	Applications	Specics	Specificity	Suppliers	Catalog Number	Dilution Ratio
ACTA2	WB,IF	Rabbit	H,M,R	Proteintech	14395-1-AP	1:2000(WB); 1:100(IF)
Akt	WB	Rabbit	H,M,R	Cell Signaling Technology	#4691	1:1000
Col 1A1	WB,IF	Rabbit	H,M,R	Proteintech	14695-1-AP	1:2000(WB); 1:50(IF)
E-Cad	WB	Rabbit	H,M,R	Proteintech	20874-1-AP	1:5000
Fibronectin	WB,IF	Rabbit	H,M,R	Proteintech	15613-1-AP	1:2000(WB); 1:50(IF)
GAPDH	WB	Rabbit	H,M,R	Cell Signaling Technology	#2118	1:1000
JNK	WB	Rabbit	H,M,R	Cell Signaling Technology	#9252	1:1000
p-Akt	WB	Rabbit	H,M,R	Cell Signaling Technology	#4060	1:1000
p-JNK	WB	Rabbit	H,M,R	Cell Signaling Technology	#4668	1:1000
p-Smad2	WB	Rabbit	H,M,R	Cell Signaling Technology	#3108	1:1000
Smad2	WB	Rabbit	H,M,R	Proteintech	12570-1-AP	1:2000
p-Smad3	WB	Rabbit	H,M,R	Cell Signaling Technology	#9520	1:2000
Smad3	WB	Mouse	H,M,R	Proteintech	66516-1-Ig	1:2000
Smad7	WB	Rabbit	H,M,R	Proteintech	25840-1-AP	1:2000
TGF-βRI	WB	Rat	H,M	R&D	AF3025	1:1000
